# Treatment outcomes of re-irradiation using stereotactic ablative radiotherapy to lung: a propensity score matching analysis

**DOI:** 10.1186/s13014-021-01948-6

**Published:** 2021-11-18

**Authors:** Tae Hoon Lee, Dong-Yun Kim, Hong-Gyun Wu, Joo Ho Lee, Hak Jae Kim

**Affiliations:** 1 Department of Radiation Oncology, Seoul National University College of Medicine and Hospital, 101 Daehak-ro, Jongno-gu, Seoul, 03080 Republic of Korea; 2 Cancer Research Institute, Seoul National University College of Medicine, 101 Daehak-ro, Jongno-gu, Seoul, 03080 Republic of Korea; 3 Institute of Radiation Medicine, Medical Research Center, Seoul National University, 101 Daehak-ro, Jongno-gu, Seoul, 03080 Republic of Korea

**Keywords:** Lung cancer, Metastasis, Stereotactic radiation therapy, Re-irradiation

## Abstract

**Background:**

The purpose of this study was to compare the treatment efficacy and safety of re-irradiation (re-RT) using stereotactic ablative radiotherapy (SABR) and initial SABR for primary, recurrent lung cancer or metastatic lung tumor.

**Methods:**

A retrospective review of the medical records of 336 patients who underwent lung SABR was performed. Re-RT was defined as the overlap of the 70% isodose line of second-course SABR with that of the initial radiotherapy, and 20 patients were classified as the re-RT group. The median dose of re-RT using SABR was 54 Gy (range 48–60 Gy), and the median fraction number was 4 (range 4–6). One-to-three case-matched analysis with propensity score matching was used, and 60 patients were included in the initial SABR group of the matched cohort.

**Results:**

The 1- and 2-year local control rates for the re-RT group were 73.9% and 63.3% and those for the initial SABR group in the matched cohort were 92.9% and 87.7%, respectively (*P* = 0.013). There was no difference in distant metastasis-free, progression-free, and overall survival rates. The crude grade ≥ 2 toxicity rates were 40.0% for the re-RT group and 25.0% for the initial SABR group (*P* = 0.318). Re-RT group had higher acute grade ≥ 2 toxicity rates (25.0% vs 5.0%, *P* = 0.031). One incident of grade 3 toxicity (pulmonary) was reported in the re-RT group; there was no grade 4‒5 toxicity.

**Conclusions:**

The local control rate of the in-field re-RT SABR was lower than that of the initial SABR without compromising the survival rates. The toxicity of re-RT using SABR was acceptable.

## Introduction

Stereotactic ablative radiotherapy (SABR) is a precise modality to deliver a high radiation dose to the tumor and is more effective than conventional radiotherapy (RT) in terms of the local control of early stage lung cancer [1]. Furthermore, studies have shown that salvage SABR can achieve acceptable local control in recurrent lung cancer after previous surgery or RT [2, 3]. Patients with oligometastatic lung tumors may have longer progression-free survival and extended time intervals between subsequent treatment interventions with SABR [4, 5]. As SABR was proven to be feasible in various clinical situations, several attempts have been made to apply SABR to previously irradiated lung lesions [6, 7]. Re-irradiation (Re-RT) has a higher risk of toxicity than initial irradiation as irreversible normal tissue damage may occur after the re-RT of the lung tumor [8]. Treatment efficacy of re-RT also needs to be considered as these previously irradiated tumors have a higher probability of developing resistance to radiation. Therefore, although the application of SABR to patients without any previous history of thoracic irradiation is feasible, the safety and efficacy of re-RT using SABR need to be verified.

Various treatment outcomes and toxicity profiles of re-RT with SABR have been reported due to heterogeneous clinical settings. The 2-year local control rates of salvage re-RT using SABR after prior external beam RT ranged from 37 to 90%, and the rates of grade 2‒3 radiation-induced lung toxicity ranged from 0 to 100% [9]. Some studies have shown considerable risk of fatal toxicity, such as pneumonitis and hemoptysis, after re-RT SABR [10]. These reports were mostly derived from retrospective single-arm studies, which made it difficult to assess the feasibility of re-RT with SABR. Therefore, this study aimed to evaluate the benefits and risks of re-RT using SABR with respect to in-field recurrence after previous irradiation of lung nodule(s) in patients with primary, recurrent lung cancer or metastatic lung tumor.

## Methods

### Study population

A retrospective review was performed of the medical records of 336 patients who underwent lung SABR for primary, recurrent lung cancer or metastatic lung tumor from January 2013 to December 2018 at a single institution. Re-RT was defined as the overlap of the 70% isodose line of second-course SABR with that of the initial RT, and 20 patients were classified as the re-RT SABR group. The remaining 316 patients were classified as the initial SABR group of the unmatched cohort.

### Treatment and follow-up

Re-RT SABR was applied when recurrence was histologically confirmed or strongly suspected in positron emission tomography / computerized tomography (PET/CT) scan and serial CT scan in the previously irradiated or adjacent areas. Patients who received re-RT SABR did not have other systemic recurrence which required chemotherapy or targeted therapy. In addition, the patients were not eligible for salvage operation due to poor lung function or refusal to surgery according to the patients’ will. Recurrent lung nodules directly abutting critical mediastinal structures such as trachea, main bronchus, great vessels and esophagus were considered not to be amenable for Re-RT SABR.

**Fig. 1:**
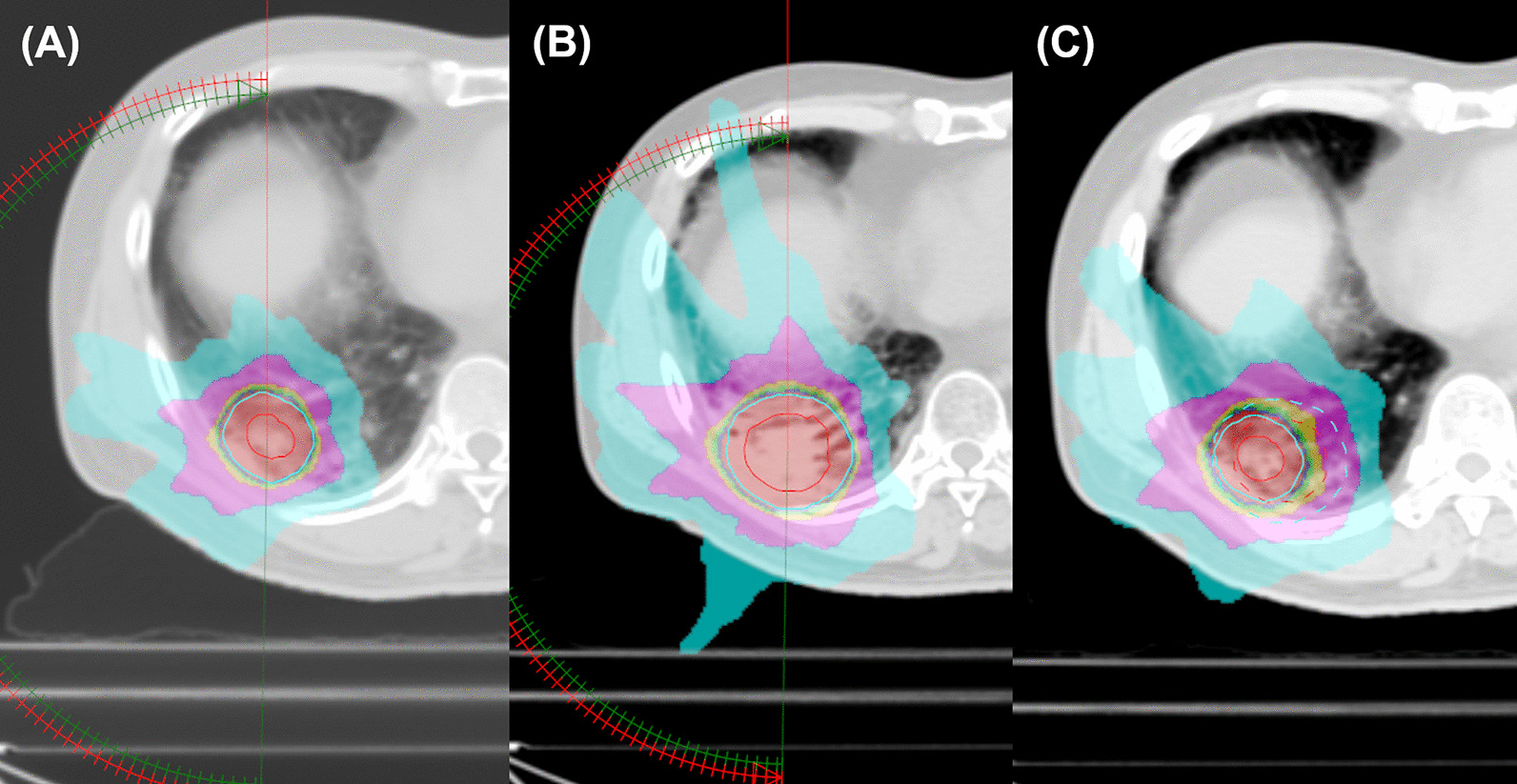
An example of re-irradiation SABR planning. **A** The patient underwent SABR for non-small-cell lung cancer with 54 Gy in 4 fraction. **B** Local recurrence was suspected one year after initial SABR, and re-irradiation SABR was delivered with 60 Gy in 4 fraction. **C** Summation of both SABR plan. Red lines are indicating internal target volume, while cyan lines are indicating planning target volume

For simulation CT scan, the patients were in the supine position with both arms abducted and immobilized by wing board and vacuum cushions. If the patient had a lung tumor in the apex of the lung, both arms were adducted, and the patient was immobilized by a thermoplastic aquaplast. An abdominal compression device was routinely applied to minimize the respiratory movement of the tumor. A four-dimensional CT (4D-CT) scan was performed for RT simulation to cover all the respiratory phases. Internal target volume (ITV) was delineated for the lung tumor, using each respiratory phase and a maximum-intensity projection image from the 4D-CT. The planning target volume (PTV) was constructed by expanding the ITV by 3‒7 mm. The prescribed dose and PTV margins were defined at the discretion of the radiation oncologists by considering various clinical factors such as tumor histology, location, baseline lung function, and previous irradiation in case of re-RT SABR. The plan was optimized to cover 95% of the PTV by 100% of the prescribed dose. Maximum dose was limited to 110%, but the PTV coverage and normal organ doses had priority over maximum dose, and maximum dose up to 113% was permitted in some cases. The treatment was delivered using the volumetric-modulated arc therapy (VMAT) technique with two 180° arcs of 6 MV photon beam in the most cases. An example of re-RT SABR planning using VMAT is illustrated in Fig. 1. In the re-RT SABR group, 2^nd^ RT course of three (15.0%) patients was delivered by magnetic resonance imaging (MRI)-guided cobalt-60 device with static intensity-modulated radiation therapy (IMRT) technique. A fraction was delivered two or three times a week, without consecutive daily treatment. For every fraction, cone beam CT was applied to verify the patient’s setup and the target location.

The patients were followed up with clinical examination and chest CT scans at 6–8 weeks after RT. Thereafter, the patients were advised to follow up with 3-month intervals for 2 years. For the third to fifth years, the recommended follow-up interval was 6 months.

### Propensity score matching

The propensity score (PS) was estimated by using the logistic regression model. Factors used in PS calculation were as follows: age (older than 75 versus younger than or equal to 75), tumor histology (lung cancer primary versus others), the existence of underlying pulmonary disease, tumor size (larger than 2 cm vs smaller than or equal to 2 cm), and tumor location (upper and middle lobes versus lower lobe). The PS-matched cohort was constructed using a 1:3 (re-RT SABR group / initial SABR group) ratio, and 60 patients from the unmatched cohort were included in the initial SABR group of the matched cohort. The standardized mean difference (SMD) was calculated to confirm the balance of the matching factors of the matched cohort. In the unmatched cohort, the SMD values of the underlying pulmonary disease and tumor size were larger than 0.2, whereas the SMD values of all variables were equal or smaller than 0.120 in the matched cohort, indicating that the matching factors became fairly balanced after PS matching. The comparisons of the matching factors of the unmatched and matched cohorts are summarized in Table 1.

**Table 1:** Comparison of the matching factors of the unmatched and matched cohorts

Characteristics	Re-irradiation SABR group (N = 20)	Initial SABR group (unmatched cohort) (N = 316)			Initial SABR group (matched cohort) (N = 60)		
		Number	*P*-value*	SMD*	Number	*P*-value*	SMD*
Age (> 75 years)	9 (45.0%)	135 (42.7%)	1.000	0.046	24 (40.0%)	0.896	0.101
Lung cancer primary	15 (75.0%)	222 (70.3%)	0.842	0.107	48 (80.0%)	0.875	0.120
Underlying pulmonary disease	8 (40.0%)	81 (25.6%)	0.265	0.302	24 (40.0%)	1.000	< 0.001
Tumor size (> 2 cm)	11 (55.0%)	133 (42.1%)	0.369	0.261	33 (55.0%)	1.000	< 0.001
Tumor location (lower lobe)	9 (45.0%)	142 (44.9%)	1.000	0.001	27 (45.0%)	1.000	< 0.001

*Compared with re-irradiation SABR group

SABR, stereotactic ablative body radiotherapy; SMD, standardized mean difference

### Clinical outcomes and toxicity profile

The clinical outcomes in this study were local control (LC), distant metastasis-free survival (DMFS), progression-free survival (PFS), and overall survival (OS), which were estimated using the Kaplan–Meier method. These treatment outcomes were measured from the date of the end of RT for each defined event. An LC event was defined as the progression of a lung tumor treated by SABR, while a DMFS event was defined as an occurrence of distant metastasis or death. A new contralateral lung nodule was considered as distant metastasis. For OS, an event was defined as the death of the patient, whereas for PFS, an event was defined as disease progression or death. Survival data were retrieved from the resident registration system of the government of the Republic of Korea. These clinical outcomes of the re-RT and initial SABR groups of the matched cohort were compared using the log-rank test. Univariate analysis was performed for LC, DMFS, PFS, and OS of the re-RT SABR group to identify potential prognostic factors affecting treatment outcomes of re-RT. Multivariate analysis was not performed due to small number of the events in the re-RT SABR group.

For the analysis of RT toxicity, new occurrences or worsening of the symptoms were recorded and graded using the Common Terminology Criteria for Adverse Events (CTCAE) version 5. Toxicity that occurred more than 3 months after the completion of RT was considered late toxicity, while other events were considered acute toxicity. The rate of freedom from grade ≥ 2 toxicity (FFT) was calculated using the Kaplan–Meier method. Univariate analyses were performed for the FFT of the re-RT SABR group using Cox proportional-hazards model.

For dosimetric analysis of organs-at-risk (OARs) for re-RT SABR group, simulation CT scans, structures, and dose distributions of 17 patients transferred from Eclipse (Version 13.6, ARIA Oncology Information System, Varian Medical Systems, Palo Alto, CA) to MIM (Version 6.1.7, MIM Software Inc., Beachwood, OH, USA). Three (15.0%) patients who were treated by MRI-guided cobalt-60 device were excluded, as dose distribution of at least one course of RT could not be retrieved. Dose distributions were converted to dose delivered in 2 Gy fractions (EQD2) using linear-quadratic model with α/β ratio of 3. Two simulation CT scans were fused by rigid assisted alignment offered by MIM, and EQD2 distribution of the first RT course was transferred to simulation CT scan of the second RT course. Summation of the two EQD2 distributions was performed, and dosimetric parameters of OAR were calculated using structures defined at the second RT course. OARs included in the analysis were lung, proximal bronchial tree (from trachea to lobar bronchus), chest wall, aorta, esophagus, and heart. OAR structure for lung was defined as lung subtracting the PTV of re-RT SABR plan. Univariate analyses were performed for pulmonary symptom grade ≥ 2 with dosimetric parameters of lung and proximal bronchial tree, and for chest wall pain grade ≥ 2 with dosimetric parameters of chest wall using logistic regression model. All statistical analyses were performed using R version 4.0.4 (The R Foundation for Statistical Computing, Vienna, Austria).

## Results

### Patient characteristics and details of re-RT SABR

The characteristics of the patients included in both groups of the matched cohort are summarized in Table 2. The median follow-up of all patients of the matched cohort was 28.0 months (range 3.5–95.8 months). The median ages of the entire matched cohort, re-RT SABR group, and the initial SABR group of the matched cohort were 70 years (range 32–85 years), 73 years (range 51–85 years), and 67.5 years (range 32–85 years), respectively. The patients were predominantly male (80.0%). About one-third of the patients had a history of pulmonary disease: three patients had interstitial lung disease (ILD), five patients had chronic obstructive pulmonary disease (COPD) (two patients were diagnosed both ILD and COPD), one patient had asthma, and one patient had a 30-year history of working at stone quarry. The median age-adjusted Charlson comorbidity index was 7 (range 3–12). Among all the matched patients, 70.0% had a history of smoking, and the median pack-year value of current and previous smokers was 40 (range 3–110 pack-years). Central lung nodules were defined as nodules closer than 2 cm from the proximal bronchial tree [11]; 14 (17.5%) patients had central nodules. 80% of the patients underwent SABR due to primary lung cancer, including both non-small-cell lung cancer and small-cell lung cancer. Among non-small-cell lung cancer patients in the re-RT SABR group, 9 patients had squamous cell carcinoma, and 5 patients had adenocarcinoma. One patient had non-small-cell lung cancer whose subtype could not be determined due to insufficient tumor tissue.

**Table 2:** Patient characteristics of the matched cohort

Characteristics	Re-irradiation SABR group (N = 20)	Initial SABR group (N = 60)	*P*-value
*Age*			0.896
≤ 75 years	11 (55.0%)	36 (60.0%)	
> 75 years	9 (45.0%)	24 (40.0%)	
*Gender*			1.000
Male	16 (80.0%)	48 (80.0%)	
Female	4 (20.0%)	12 (20.0%)	
*Underlying pulmonary disease*			1.000
No	12 (60.0%)	36 (60.0%)	
Yes	8 (40.0%)	24 (40.0%)	
*Age-adjusted Charlson Comorbidity Index*			1.000
< 10	18 (90.0%)	55 (91.7%)	
≥ 10	2 (10.0%)	5 (8.3%)	
*Smoking history*			0.778
No	5 (25.0%)	19 (31.7%)	
Yes	15 (75.0%)	41 (68.3%)	
*Current smoking*			1.000
No	16 (80.0%)	50 (83.3%)	
Yes	4 (20.0%)	10 (16.7%)	
*Lung operation history*			1.000
No	15 (75.0%)	44 (73.3%)	
Yes	5 (25.0%)	16 (26.7%)	
*Location*			0.897
Upper and middle lobe	10 (50.0%)	33 (55.0%)	
Lower lobe	10 (50.0%)	27 (45.0%)	
*Centrality*			0.497
Peripheral	15 (75.0%)	51 (85.0%)	
Central	5 (25.0%)	9 (15.0%)	
*Size*			0.897
≤ 2 cm	10 (50.0%)	27 (45.0%)	
> 2 cm	10 (50.0%)	33 (55.0%)	
*Histology*			0.218
NSCLC	15 (75.0%)	45 (80.0%)	
SCLC	1 (5.0%)	0 (0.0%)	
Others	4 (20.0%)	12 (20.0%)	

SABR, stereotactic ablative body radiotherapy; NSCLC, Non-small-cell lung cancer; SCLC. Small-cell lung cancer

The details of re-RT SABR are summarized in Table 3. The median prescribed dose of re-RT SABR was 54 Gy (range 48–60 Gy), and all but one patient had 4 fractionations. For the initial SABR group of the matched cohort, the median prescribed dose was 60 Gy (range 45–60 Gy), and the median fractionation number was 4 (range 4–8). Most (88.3%) of the patients in the initial SABR group underwent SABR with 4 fractionations. There was no significant difference in the prescribed doses for both groups (*P* = 0.342). The median biologically equivalent dose with an α/β ratio of 10 (BED_10_) was 126.9 Gy (range 105.6–150.0 Gy) for the re-RT SABR group and 126.9 Gy (range 85.5–150.0 Gy) for the initial SABR group. There was no significant difference in the BED_10_ values of both the groups (*P* = 0.461). Only one patients from the initial SABR group received a BED_10_ lower than 100 Gy. For the re-RT SABR group, the median cumulative BED_10_ of two courses of irradiation was 262.6 Gy (range 184.8–300.0 Gy).

**Table 3:** Radiotherapy of re-irradiation SABR

Parameters	Value
Interval to re-irradiation (months, median, range)	13.8 (2.0–51.6)
Dose (Gy, median, range)	54 (48–60)
*Fractionations*	
4	19 (95.0%)
6	1 (5.0%)
Dose, BED_10_ (Gy, median, range)	126.9 (105.6–150.0)
*Any treatment between RTs*	
No	17 (85.0%)
Yes	3 (15.0%)
*Previous RT technique*	
3D-CRT	6 (30.0%)
SABR	14 (70.0%)
Median ITV (mL, median, range)	6.0 (0.6–26.7)
Median PTV (mL, median, range)	22.7 (3.4–77.1)

SABR, stereotactic ablative body radiotherapy; BED_10_, biologically equivalent dose with α/β of 10; 3D-CRT, 3-dimensional conformal radiation therapy; ITV, internal target volume; PTV, planning target volume

For re-RT SABR, the prescribed dose was significantly different from the centrality of the lung nodule (*P* = 0.039). The median doses for peripheral and central nodules were 57 Gy (range 48–60 Gy) and 52 Gy (range 48–54 Gy), respectively. The BED_10_ value was not significantly different (*P* = 0.063) for both the groups: the median BED_10_ values were 126.9 Gy (range 105.6–150.0 Gy) for peripheral nodules and 119.6 Gy (range 105.6–126.9 Gy) for central nodules. There was no significant difference in the prescribed dose (*P* = 0.804) and BED_10_ values (*P* = 0.658) between primary lung cancer and metastatic lung tumors.

### Treatment outcomes

**Fig. 2:**
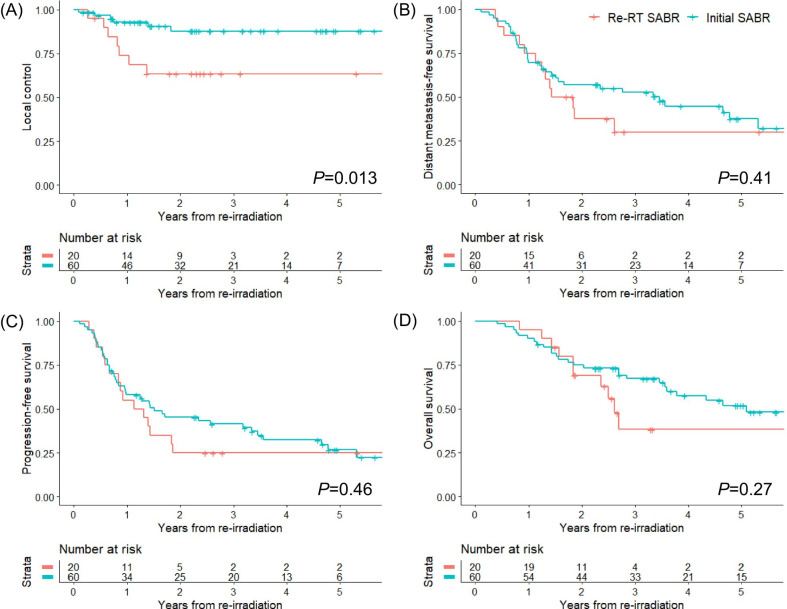
Treatment outcomes of the matched cohort. **A** Local control, **B** distant metastasis-free survival, **C** progression-free survival, **D** overall survival

The 1-, 2-, and 3-year LC rates were 73.9%, 63.3%, and 63.3% in the re-RT SABR group and 92.9%, 87.7%, and 87.7% in the initial SABR group of the matched cohort, respectively. There was a significant difference in the LC rates between the two groups (*P* = 0.013). The 1-, 2-, and 3-year DMFS rates were 75.0%, 37.5%, and 30.0% in the re-RT SABR group and 69.7%, 57.1%, and 52.8% in the initial SABR group, respectively, with no significant difference (*P* = 0.410) between both the groups. The PFS rates at 1, 2, and 3 years were 55.0%, 25.0%, and 25.0% for the re-RT SABR group and 58.0%, 45.4%, and 41.4% for the initial SABR group, respectively. No significant difference in PFS rates was found between the two groups (*P* = 0.460). The OS rates at 1, 2, and 3 years were 95.0%, 69.1% and 38.3% for the re-RT SABR group and 90.0%, 74.8%%, and 67.1% for the initial SABR group, respectively. There was no significant difference in the OS rates between the two groups (*P* = 0.270). These treatment outcomes of the matched cohort are illustrated in Fig. 2.

The results of the univariate analysis of LC, DMFS, PFS, and OS for the re-RT SABR group are summarized in Table 4. There was no significant association between these variables and LC in the univariate analysis. Also, we could not find significant factors for DMFS, although cumulative BED_10_ was marginally associated with DMFS (hazard ratio [HR] 0.315, 95% confidence interval [CI] 0.099–1.001, *P* = 0.050). The RT technique of the first course was the only factor that associated with PFS in the univariate analysis (HR 4.847, 95% CI 1.073–21.91, *P* = 0.040). In terms of OS, the RT technique of the first course (HR 9.167, 95% CI 1.062–79.08, *P* = 0.044) and any treatment between the RT courses (HR 5.686, 95% CI 1.265–25.55, *P* = 0.023) were associated.

**Table 4:** Univariate analysis of treatment outcomes of the re-irradiation SABR group

Variable (comparison vs reference)	Local control			Distant metastasis–free survival			Progression–free survival			Overall survival		
	HR	95% CI	*P* value	HR	95% CI	*P* value	HR	95% CI	*P* value	HR	95% CI	*P* value
Age (> 75 years vs ≤ 75)	1.239	0.276–5.556	0.780	1.264	0.423–3.782	0.675	1.108	0.401–3.062	0.844	3.287	0.843–12.82	0.087
Tumor size (> 2 vs ≤ 2 cm)	0.606	0.135–2.717	0.513	1.085	0.363–3.242	0.884	0.637	0.229–1.769	0.387	2.424	0.622–9.441	0.202
Location (lower lobe vs upper and middle)	0.952	0.213–4.267	0.949	0.944	0.315–2.831	0.918	0.776	0.279–2.156	0.626	2.607	0.669–10.16	0.168
Tumor centrality (central vs peripheral)	0.418	0.050–3.474	0.419	0.751	0.206–2.741	0.664	0.543	0.153–1.929	0.345	0.569	0.115–2.822	0.490
Histology (squamous cell carcinoma vs others)	4.094	0.788–21.27	0.094	0.932	0.312–2.788	0.900	1.325	0.478–3.674	0.589	1.745	0.501–6.082	0.382
Smoking (≥ 10 vs < 10 pack-year)	1.393	0.270–7.203	0.692	1.841	0.503–6.732	0.356	1.839	0.582–5.811	0.299	2.164	0.458–10.23	0.330
Concurrent chemotherapy at first RT course (yes vs no)	–	–	–	0.322	0.041–2.515	0.280	0.250	0.033–1.913	0.182	-	-	-
RT technique of first course (SABR vs 3D-CRT)	2.975	0.357–24.77	0.313	4.056	0.836–19.68	0.082	4.847	1.073–21.91	0.040	9.167	1.062–79.08	0.044
Any treatment between RT courses (yes vs no)	2.436	0.471–12.59	0.288	1.984	0.535–7.359	0.306	1.267	0.357–4.506	0.714	5.686	1.265–25.55	0.023
Treatment interval between RT courses (> 12 vs ≤ 12 months)	1.272	0.246–6.569	0.774	0.665	0.203–2.178	0.501	0.981	0.311–3.096	0.974	1.517	0.319–7.226	0.601
PTV volume (> 50 vs ≤ 50 cm^3^)	3.052	0.574–16.23	0.191	1.175	0.249–5.545	0.839	1.926	0.523–7.093	0.324	4.53	0.802–25.60	0.087
Age-adjusted Charlson Comorbidity Index (≥ 10 vs < 10)	–	–	–	0.485	0.062–3.812	0.491	0.328	0.042–2.530	0.285	0.988	0.121–8.078	0.991
Cumulative BED_10_ (≥ 120 Gy vs < 120)	2.477	0.298–20.59	0.401	0.315	0.099–1.001	0.050	0.602	0.212–1.715	0.342	0.371	0.106–1.295	0.120

SABR, stereotactic ablative body radiotherapy; HR, hazard ratio; CI, confidence interval; RT, radiotherapy; 3D-CRT, 3-dimensional conformal radiation therapy; PTV, planning target volume; BED_10_, biologically equivalent dose with α/β of 10

### Toxicity profile and dosimetric analysis

**Fig. 3:**
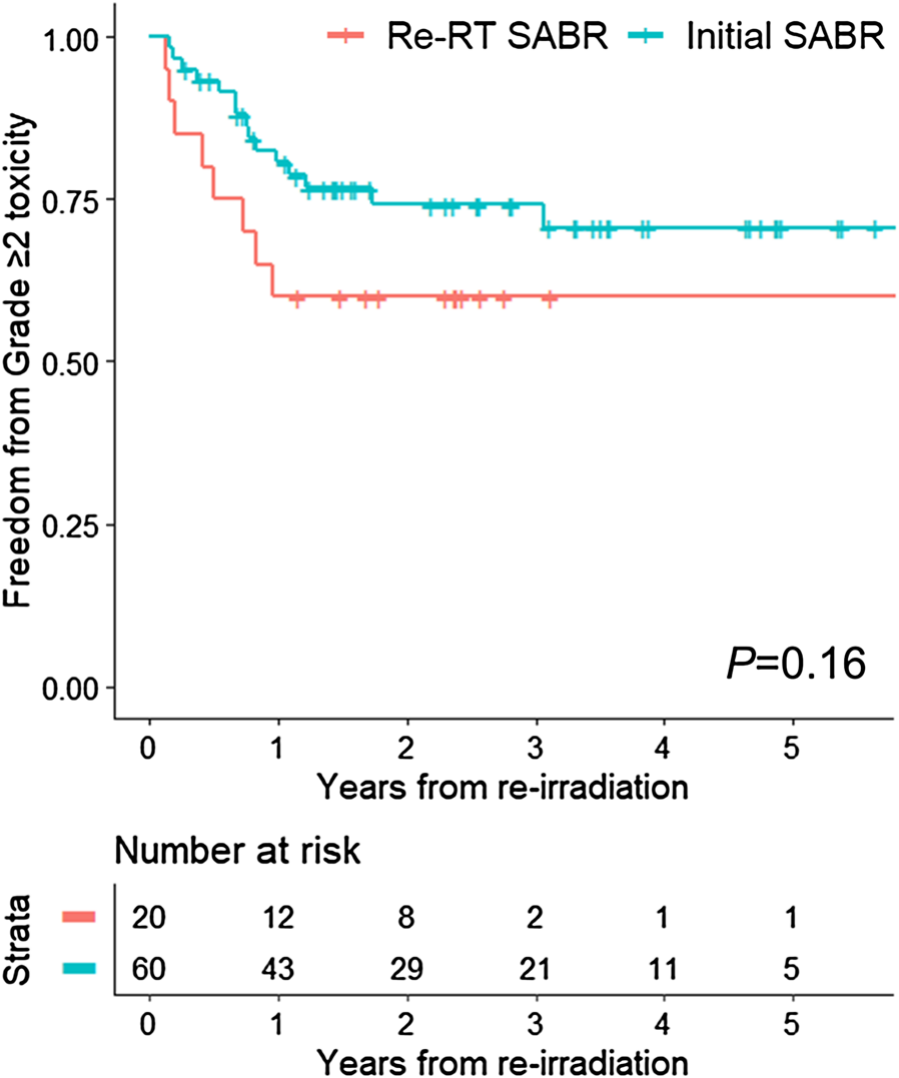
The rate of freedom from grade ≥ 2 toxicity

Toxicity profiles of the matched cohort are summarized in Table 5. The reported complications were cough, dyspnea, and chest wall pain. In the patients of the re-RT SABR group, 40.0% experienced grade ≥ 2 toxicity, while 25.0% of the patients in the initial SABR group showed grade ≥ 2 toxicity. There were no significant differences in the crude rates of grade ≥ 2 toxicity between both the groups (*P* = 0.318). More grade ≥ 2 acute toxicity was reported in the re-RT SABR group (25.0% vs 5.0%, *P* = 0.031), whereas there was no difference in the occurrence rates of grade ≥ 2 late toxicity (20.0% vs 21.7%, *P* = 1.000). The 1-, 2-, and 3-year rates of FFT were 60.0%, 60.0%, and 60.0% for the re-RT SABR group and 80.6%, 74.2%, and 74.2% for the initial SABR group, respectively. The log-rank test showed no significant difference in the FFT rates between the two groups (*P* = 0.160). FFT rates are shown in Fig. 3. The Cox univariate analysis for the re-RT SABR group revealed age-adjusted Charlson comorbidity index ≥ 10 (HR 8.302, 95% CI 2.361–50.66, *P* = 0.022), and any other treatment between the two courses of RT (HR 8.091, 95% CI 1.609–40.69 *P* = 0.011) were associated with the FFT rate.

**Table 5:** Toxicity profile of the matched cohort

Toxicities	Re-irradiation SABR group (N = 20)					Initial SABR group (N = 60)				
	Grade 1	Grade 2	Grade 3	Grade 4	Grade 5	Grade 1	Grade 2	Grade 3	Grade 4	Grade 5
*Acute*										
Cough	1 (5.0%)	3 (15.0%)	0 (0.0%)	0 (0.0%)	0 (0.0%)	3 (5.0%)	2 (3.3%)	0 (0.0%)	0 (0.0%)	0 (0.0%)
Dyspnea	1 (5.0%)	3 (15.0%)	0 (0.0%)	0 (0.0%)	0 (0.0%)	2 (3.3%)	1 (1.7%)	0 (0.0%)	0 (0.0%)	0 (0.0%)
Chest wall pain	2 (10.0%)	1 (5.0%)	0 (0.0%)	0 (0.0%)	0 (0.0%)	1 (1.7%)	0 (0.0%)	0 (0.0%)	0 (0.0%)	0 (0.0%)
*Chronic*										
Cough	0 (0.0%)	1 (5.0%)	0 (0.0%)	0 (0.0%)	0 (0.0%)	2 (3.3%)	3 (5.0%)	1 (1.7%)	0 (0.0%)	0 (0.0%)
Dyspnea	0 (0.0%)	0 (0.0%)	1 (5.0%)	0 (0.0%)	0 (0.0%)	10 (16.7%)	3 (5.0%)	2 (3.3%)	0 (0.0%)	1 (1.7%)
Chest wall pain	0 (0.0%)	2 (10.0%)	0 (0.0%)	0 (0.0%)	0 (0.0%)	2 (3.3%)	5 (8.3%)	0 (0.0%)	0 (0.0%)	0 (0.0%)

Dosimetric analysis of OARs for 17 patients is summarized in Table 6. Cumulative EQD2 for chest wall was relatively high, while cumulative EQD2 for critical mediastinal structures including proximal bronchial tree, aorta, esophagus, and heart were low. Dosimetric parameters of lung and chest wall did not show significant association with occurrence of grade ≥ 2 pulmonary toxicity (cough, dyspnea) and chest wall pain, respectively, in the univariate analysis.

**Table 6:** Dosimetric analysis of organs at risk for the re-irradiation SABR group

Organs	Parameters	Cumulative EQD2 (Gy, median, range)	Univariate analysis with grade ≥ 2 toxicity		
			OR	95% CI	*P* value
Lung (unilateral)	Mean dose	20.19 (5.16–38.88)	0.859^†^	0.724–1.019	0.081
	V20 (%)	26.79% (5.75–68.25%)	0.918^†^	0.824–1.021	0.116
Lung (bilateral)	Mean dose	11.88 (2.50–20.73)	0.824^†^	0.638–1.064	0.138
	V20 (%)	11.67% (2.59–32.52%)	0.894^†^	0.762–1.049	0.171
Proximal bronchial tree	D0.5 cc	53.86 (0.39–124.57)	0.980^†^	0.948–1.014	0.244
	D1cc	46.88 (0.35–84.57)	0.969^†^	0.931–1.009	0.128
	D5cc	17.87 (0.28–79.20)	0.950^†^	0.892–1.011	0.109
	D10cc	11.55 (0.24–73.74)	0.931^†^	0.831–1.043	0.219
	D15cc	8.56 (0.19–69.84)	0.924^†^	0.797–1.072	0.297
	D30cc	1.26 (0.11–64.57)	0.468^†^	0.098–2.228	0.340
Chest wall	D0.5 cc	199.45 (142.05–357.78)	1.024^‡^	0.998–1.051	0.066
	D1cc	179.44 (119.39–335.21)	1.024^‡^	0.997–1.050	0.077
	D5cc	131.28 (42.74–261.98)	1.021^‡^	0.995–1.047	0.114
	D10cc	110.07 (17.33–220.61)	1.024^‡^	0.995–1.055	0.110
	D15cc	97.22 (13.41–198.90)	1.028^‡^	0.994–1.063	0.103
	D30cc	75.64 (9.16–162.43)	1.033^‡^	0.994–1.074	0.096
Aorta	D0.5 cc	32.63 (6.96–233.03)	–	–	–
	D1cc	30.40 (6.45–195.23)	–	–	–
	D5cc	24.30 (5.19–99.37)	–	–	–
	D10cc	20.69 (4.69–69.04)	–	–	–
	D15cc	18.10 (4.18–66.20)	–	–	–
	D30cc	12.30 (1.54–59.15)	–	–	–
Esophagus	D0.5 cc	18.25 (6.20–63.97)	–	–	–
	D1cc	17.09 (5.70–61.28)	–	–	–
	D5cc	12.22 (1.56–50.37)	–	–	–
	D10cc	2.85 (0.32–27.04)	–	–	–
	D15cc	0.89 (0.21–18.61)	–	–	–
Heart	D0.5 cc	32.80 (0.20–93.95)	–	–	–
	D1cc	30.71 (0.20–90.38)	–	–	–
	D5cc	23.27 (0.19–79.56)	–	–	–
	D10cc	19.14 (0.17–71.93)	–	–	–
	D15cc	16.69 (0.16–70.19)	–	–	–
	D30cc	12.58 (0.15–67.12)	–	–	–

^†^Calculated with grade ≥ 2 pulmonary toxicity (cough, dyspnea)

^‡^Calculated with grade ≥ 2 chest wall pain

EQD2, dose delivered in 2 Gy fractions; OR, odds ratio; CI, confidence interval

## Discussion

The current study demonstrates that the LC rate of the salvage lung SABR after in-field recurrence is acceptable with tolerable toxicity. In-field locoregional relapse after definitive chemoradiation for locally advanced lung cancer is frequent [12]. Even with high LC rates, the local recurrence after lung SABR is not uncommon and has an actuarial rate of nearly 20% [13]. Several reports have shown that salvage surgical resection can be an alternative [14, 15]; however, surgery is not always an option as many lung cancer patients are morbid and have poor lung function. Therefore, salvage SABR is still a promising treatment option even with in-field recurrence after thoracic irradiation. Nevertheless, it is important to pay attention to potentially worse treatment outcomes of salvage SABR than those of initial irradiation, as the current study found lower LC rates for re-RT SABR by PS matching than for initial lung SABR without any previous irradiation history.

Although there have been several studies on thoracic re-RT, the definition of re-RT varies considerably. Some reports included both in-field and out-field recurrence as targets for re-RT [16], hindering the interpretation of treatment outcomes for patients with high cumulative dose using re-RT. Even in studies on only in-field recurrence, the definition of in-field recurrence is diverse. Some studies used a certain percentage or absolute isodose line [6, 17, 18], while others used criteria directly derived from the target volume [19]. Several studies used overlapping of 50% isodose line as the inclusion criteria [6, 18], but it did not guarantee the inclusion of the patients with higher cumulative dose than commonly prescribed dose when two dose distributions marginally overlap. Our definition of re-RT SABR is the overlap of the 70% isodose line to ensure that the recruited patients had a heavily irradiated area of at least 70% of the cumulative dose.

A few reports of salvage SABR for in-field lung recurrence after previous RT have been published, although the comparison between re-RT SABR and initial SABR is scarce. Wide ranges of LC rates were reported due to various clinical situations of utilization of re-RT SABR [9], making it even more difficult to compare the treatment outcomes among retrospective series. The current study applied PS matching to mitigate these clinical differences and found worse LC rates in the re-RT SABR group. The clinical outcomes reported by the current study would be helpful to assess the actual clinical benefits of re-RT using SABR in settings of in-field local recurrence. Moreover, in contrast to some previous re-RT SABR series, all second-course SABR in the current study comprised irradiation with high doses of more than 100 Gy of BED_10_, which was considered sufficient to achieve LC of lung nodules [10, 17, 20, 21]. This indicated that even with a sufficient dose, the LC rate of re-RT SABR for in-field recurrent lung nodules may be lower than that of initial SABR.

There are several reasons for low LC of locally relapsed nodules treated with re-RT SABR. One plausible explanation is that surviving cell fractions that cause local recurrence after RT are radioresistant, and underlying mechanisms have been postulated for this phenomenon [22]. Additionally, there is a chance that locally recurrent lung nodules could initially have radioresistant clones. Radiation lung fibrosis may impact tissue oxygenation, and impaired oxygenation could reduce radiosensitivity [23]. Delineating target volume in re-RT SABR may be inaccurate due to radiation pneumonitis after initial RT. The exact reasons of low LC need to be addressed by further study.

Toxicity is an important concern of re-RT, as cumulative radiation damage would result in a greater risk of damage to normal tissue. Over 40% of the patients from the re-RT group of the current study experienced grade ≥ 2 toxicity. However, only one grade 3 toxicity was reported, and there was no grade 4‒5 toxicity. The low rate of severe toxicity of re-RT SABR in the current study might be influenced by the utilization of advanced RT technology such as VMAT. Most (85.0%) re-RT SABR courses in this study were delivered by using the VMAT technique in contrast to other studies in which the patients underwent re-RT SABR by three-dimensional conformal RT or static IMRT. Nevertheless, this result should be interpreted cautiously, as the possibility of severe and fatal toxicity of re-RT SABR cannot be excluded due to the small sample size and the retrospective nature of this study. Some studies have demonstrated high rates of severe toxicity. For instance, a re-RT SABR study by Peulen et al. [19] showed that 37.9% of the patients experienced grade ≥ 3 toxicity with 3 fatal cases. The centrality of the recurrent lung nodules was not a significant risk factor of occurrence of grade ≥ 2 toxicity in the current study. However, additional caution would be needed for central tumors, as several fatal pneumonitis and hemoptysis were reported in previous series [6, 10]. It should be noted that although there were lesions classified as central by the present criteria, no central lung nodules in re-RT SABR group of this study had direct abutment to the trachea, esophagus, or the main bronchus. Central nodules were re-irradiated only when a safe SABR plan could be executed.

There are several different methods for reflecting dose distribution of previous RT course when planning re-RT SABR. Cumulative EQD2 based on linear quadratic equation was applied in a recently published article [24]. As we previously mentioned, dosimetric analysis in the current study showed relatively low cumulative EQD2 for critical mediastinal structures when compared with that of chest wall, reflecting patient selection factor. No significant association between dosimetric parameters and occurrence of grade ≥ 2 toxicity was observed, presumably due to the small number of the event. Since some dosimetric parameters of the current series showed marginal significance, it is expected that a significant dose-response relationship can be confirmed through a larger number of patient studies.

The current study has some limitations. As this series included both primary lung cancer and metastatic lung tumors, various histologic types were included in the analysis, which might have influenced the treatment outcomes due to differences in radioresistance based on tumor histology. Second, the definition of re-RT used in this study was arbitrary, and both conventional fractionation RT and SABR were allowed as first-course RT in the re-RT SABR group, creating a lack of uniformity in the clinical situations. Third, the patients might not be representative due to the small numbers of the recruited cases. It should be considered that it would be impractical to design a prospective trial for re-RT SABR to recurrent lung nodules, as recurrence after initial SABR to lung is not common due to high local control rate of SABR. Therefore, we conducted propensity-score matching analysis using retrospective data. Finally, toxicity could be under-reported due to the retrospective nature of this study. Nevertheless, the results of the present study are clinically significant because it included only those patients who underwent re-RT for in-field recurrence in a strict sense. In terms of the heterogeneous inclusion of histologic types and techniques of the first course of RT, despite the inherent bias in a retrospective study, this study captured complex real-world situations of re-RT by dealing with various cases. Further, the patients in the re-RT SABR group underwent SABR with a more advanced RT technique and had higher BED_10_ values than those in previous reports. Furthermore, as reports comparing re-RT SABR to initial SABR are rare, this study can contribute to clinical decisions for the treatment of recurrent lung nodules.

## Conclusions

Re-RT SABR for in-field local recurrence after thoracic irradiation is effective and feasible, although the LC rate for in-field re-RT SABR was lower than for initial SABR. Albeit only one grade 3 toxicity and no grade 4‒5 toxicity was reported in the re-RT SABR group, it should be noted that patients without direct abutment to critical mediastinal structures underwent re-RT SABR in the current study. The biological background of the worse outcome needs to be further explored in future studies. Even though the toxicity of re-RT SABR was acceptable, more clinical data would be needed to specify the criteria for safe re-RT.

## Data Availability

The datasets used and/or analysed during the current study are available from the corresponding author on reasonable request.

## Abbreviations

RT: Radiotherapy
Re-RT: Re-irradiation
SABR: Stereotactic ablative radiotherapy
PET/CT: Positron emission tomography/computerized tomography
4D-CT: Four-dimensional computerized tomography
ITV: Internal target volume
PTV: Planning target volume
VMAT: Volumetric-modulated arc therapy
MRI: Magnetic resonance imaging
IMRT: Intensity-modulated radiation therapy
PS: Propensity score
SMD: Standardized mean difference
LC: Local control
DMFS: Distant metastasis-free survival
PFS: Progression-free survival
OS: Overall survival
CTCAE: Common Terminology Criteria for Adverse Events
FFT: Freedom from grade ≥ 2 toxicity
OARs: Organs-at-risk
EQD2: Dose delivered in 2 Gy fractions
ILD: Interstitial lung disease
COPD: Chronic obstructive pulmonary disease
BED_10_: Biologically equivalent dose with an α/β ratio of 10
HR: Hazard ratio
CI: Confidence interval
